# Preventing pediatric chronic postsurgical pain: Time for increased rigor

**DOI:** 10.1080/24740527.2021.2019576

**Published:** 2022-04-28

**Authors:** Christine B. Sieberg, Keerthana Deepti Karunakaran, Barry Kussman, David Borsook

**Affiliations:** a Biobehavioral Pediatric Pain Lab, Department of Psychiatry & Behavioral Sciences, Boston Children’s Hospital, Boston, Massachusetts, United States; b Pain and Affective Neuroscience Center, Department of Anesthesiology, Critical Care, and Pain Medicine, Boston Children’s Hospital, Boston, Massachusetts, United States; c Department of Psychiatry, Harvard Medical School, Boston, Massachusetts, United States; d Department of Anesthesiology, Critical Care, & Pain Medicine, Boston Children’s Hospital, Boston, Massachusetts, United States; e Department of Anesthesiology, Harvard Medical School, Boston, Massachusetts, United States; f Department of Psychiatry and Radiology, Massachusetts General Hospital, Hospital, Harvard Medical School, Boston, Massachusetts, United States

**Keywords:** chronic pain, surgery, postoperative pain, biomarker, fNIRS, cognitive behavioral therapy, analgesia, opioids

## Abstract

Chronic postsurgical pain (CPSP) results from a cascade of events in the peripheral and central nervous systems following surgery. Several clinical predictors, including the prior pain state, premorbid psychological state (e.g., anxiety, catastrophizing), intraoperative surgical load (establishment of peripheral and central sensitization), and acute postoperative pain management, may contribute to the patient’s risk of developing CPSP. However, research on the neurobiological and biobehavioral mechanisms contributing to pediatric CPSP and effective preemptive/treatment strategies are still lacking. Here we evaluate the perisurgical process by identifying key problems and propose potential solutions for the pre-, intra-, and postoperative pain states to both prevent and manage the transition of acute to chronic pain. We propose an eight-step process involving preemptive and preventative analgesia, behavioral interventions, and the use of biomarkers (brain-based, inflammatory, or genetic) to facilitate timely evaluation and treatment of premorbid psychological factors, ongoing surgical pain, and postoperative pain to provide an overall improved outcome. By achieving this, we can begin to establish personalized precision medicine for children and adolescents presenting to surgery and subsequent treatment selection.

## RÉSUMÉ

La douleur chronique post-chirurgicale (DCPC) résulte d'une cascade d'événements dans les systèmes nerveux central et périphérique suite à une intervention chirurgicale. Plusieurs prédicteurs cliniques, y compris l'état douloureux antérieur, l'état psychologique prémorbide (p. ex., anxiété, catastrophisme), la charge chirurgicale peropératoire (établissement d'une sensibilisation périphérique et centrale) et la prise en charge de la douleur postopératoire aiguë, peuvent contribuer au risque du patient de développer une DCPC. Cependant, la recherche sur les mécanismes neurobiologiques et biocomportementaux contribuant à la DCPC pédiatrique et sur les stratégies de prévention et de traitement efficaces font encore défaut. Nous évaluons ici le processus périchirurgical en cernant les problémes clés et en proposant des solutions potentielles pour les états douloureux pré, per et postopératoires afin de prévenir et de prendre en charge la transition de la douleur aiguë à la douleur chronique. Nous proposons un processus en huit étapes impliquant l'analgésie préemptive et préventive, les interventions comportementales et l'utilisation de biomarqueurs (cérébraux, inflammatoires ou génétiques) pour faciliter l'évaluation et le traitement opportuns des facteurs psychologiques prémorbides, de la douleur chirurgicale persistante et de la douleur postopératoire afin d'améliorer le résultat global. En y parvenant, nous pouvons commencer à établir une médecine de précision personnalisée pour les enfants et les adolescents qui subissent une intervention chirurgicale et à la sélection du traitement qui s'ensuit.

## Introduction

Acute pain following surgery can occur immediately after surgery to up to 3 months postoperation.^1^ Progression to moderate or severe chronic postsurgical pain (CPSP; pain lasting longer than 2 months postsurgery)^2^ occurs in 10% to 30% of patients, with 25% of adults referred to chronic pain clinics identifying surgery as the antecedent.^3–6^ A number of clinical predictors likely contribute to pain chronification, such as intraoperative pain load, pre- and postoperative pain intensity, hyperalgesia/allodynia,^7^ as well as other biopsychosocial factors, including psychological distress (e.g., anxiety, depression, catastrophizing),^2,4,8^ and biological (e.g., age, pain sensitivity, genetics)^9–11^ and social (e.g., poorly treated postoperative pain) factors, a complex combination of factors resulting in central sensitization.^12,13^

There are far-reaching consequences for quality of life and physical and emotional functioning for those affected by CPSP, with one alarming consequence being the opioid epidemic.^14^ Though the nature and extent of the epidemic and the degree to which it relates to CPSP varies in different populations, health care settings, and countries, opioid use and subsequent abuse are a consequence of living with CPSP.^15,16^ For example, though opioid prescriptions after major surgery are often unavoidable, one study found that adults who were opioid-naïve prior to undergoing a minor surgery still had a 7.7% chance of continued opioid use 1 year postsurgery.^17^ Additionally, though the United States has an alarming rate of opioid use, abuse, overdoses, and deaths, with the number of people dying from opioid overdose increasing by 120% between 2010 and 2018, other countries have not been immune. It is estimated that there are approximately 53 million people (adolescents and adults) globally who use opioids, with close to 400,000 deaths annually attributed to opioids.^18^ Yet despite these high prevalence rates and negative impact, research on the mechanisms contributing to CPSP, as well as effective treatment strategies, is lacking. This is surprising given that aside from premorbid pain syndromes (including rare disease; chronic remitting conditions), surgery provides an ideal “experimental” condition where the timing of the intervention is known and thus presurgical, intrasurgical, and postsurgical processes can be evaluated or defined. Though these issues are well noted in the adult population, relatively little research in these domains is reported in children (i.e., individuals <18 years of age).

With over 6 million pediatric surgeries performed yearly in the United States alone,^19^ a better understanding of CPSP in children warrants further investigation. Children provide a unique perspective on the evolution of chronic pain after surgery. Overall, most children are reported to be relatively resistant to development of CPSP, with prevalence rates for CPSP in children, especially very young children, seemingly low^20^; however, this is not always the case. For a subset of youth, the experience of CPSP may be compounded by the fact that the resultant effects on synaptic plasticity during critical developmental stages may persist into adulthood.^21,22^ As with the adult experience, CPSP is further complicated by research showing an association between medical use of prescribed opioids during adolescence and later nonmedical opioid use in adulthood.^23,24^

Though factors contributing to CPSP in adults have been studied and include risks such as presurgical pain levels,^7^ the surgery itself^7^ (e.g., surgery duration, intraoperative nerve injury), and psychosocial (e.g., pain catastrophizing,^4^ pre- and postoperative depression,^2^ anxiety,^8^ functional disability^8^) and biological (e.g., psychophysical pain sensitivity,^11^ poor diffuse noxious inhibitory control efficiency^25^) factors, there are substantial gaps in our understanding of the unique drivers of *pediatric* CPSP. A better understanding of the mechanisms contributing to CPSP will allow for the establishment of personalized precision medicine for patients presenting to surgery and subsequent treatment selection. The ultimate goal should be to implement effective peri- and postsurgical interventions and to utilize biomarkers to ameliorate the progression of pain from acute to chronic.

**Figure 1.**
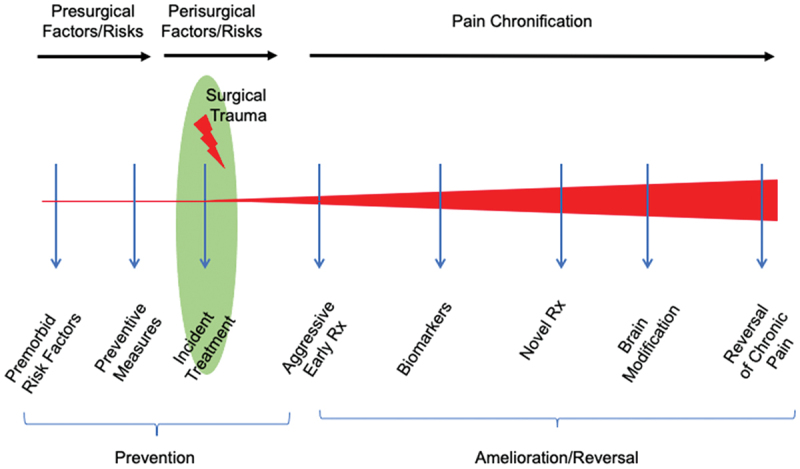
Proposed sequential process in preventing and treating chronic pain in the surgical patient. The program encompasses a process that involves ongoing and continuous evaluation and treatment of premitigating factors to premorbid status, injury and immediate postinjury treatments (including perisurgical processes), objective assessment of pain chronification, and treatment rehabilitative processes.

Here we review the perisurgical process, in terms of adopting processes that may provide improved outcomes. Specifically, we identify key problems and propose potential solutions for three states: (1) pre-, (2) intra, and (3) postoperative pain, based on our previously published work,^26^ which provided an immediate and continuous framework for evaluating the natural history (evolution, progression) of CPSP. Importantly, this model involves ongoing and continuous evaluation and treatment of premitigating factors to premorbid status, injury, and immediate postinjury treatments (including perisurgical processes); objective assessment of pain chronification; and treatment rehabilitative processes. We model these processes in Figure 1.

## State I: Presurgical

### Current Problem

The presurgical period affords an opportunity to define the risk status of a surgical candidate to developing CPSP; however, this does not occur. Though there are strong data in adults to support the notion that presurgical factors such as psychological state,^2,4,8^ pain level,^7^ and functioning of the endogenous pain modulation systems^11,25^ can predict postsurgical outcomes, these variables have rarely been studied in pediatric patients, and very few of these are systematically applied in routine clinical practice to prevent the development of CPSP. Additionally, the prior brain state may confer resilience or susceptibility to the evolution of postsurgical chronic pain; however, the individual or cumulative (interactive) effects on brain systems are unknown. Hence, there is a need to identify biomarkers that can predict and prevent pain chronification and to understand how biomarkers interact with emotional and neurological functioning to impact surgical pain outcomes.

Genetic and immunological biomarkers are likely important in predicting who is at risk for developing CPSP, but there are substantial gaps in our understanding of how these factors specifically confer risk. A recent meta-analysis and systematic review of 21 studies evaluating genetic risk for CPSP in adults concluded that six variants (five genes) marginally increased risk for CPSP associated with rs734784 A>G of the potassium voltage-gated channel gene (*KCNS1*).^27^

Additionally, recent research from our group found differential expression of genes for the human leukocyte antigen complex and genes regulating chemokine receptors between pediatric patients who developed CPSP versus those who did not.^9^ These findings are consistent with other studies on adults implicating the human leukocyte antigen complex in several chronic pain states^28,29^ and suggest that uncovering the underlying mechanisms of the CPSP transition may lie in understanding the innate immune response to surgical procedures. Other recent work from our group on adolescent patients found alterations in micro-RNA expression in multiple cellular stress and inflammation pathways after peripheral nerve injury, as well as changes on multimodal assessment, including functional magnetic resonance imaging (fMRI) and psychological measures, suggesting that micro-RNA changes may be linked to both the peripheral nervous system and changes in the brain, emotional functioning, and neuroinflammatory signaling pathways.^30^ This cascade model would likely be useful to apply to a surgical model.

In addition to the identification of biomarkers that may contribute to pediatric CPSP and their interaction with psychosocial and neural mechanisms, there is a problem with how potential risks are evaluated clinically prior to surgery. Though some of this can be attributed to a lack of understanding of the mechanisms, such as the aforementioned biomarkers, that contribute to CPSP, we are not aware of any widely used routine application of a validated screening tool assessing for potential risk factors for the development CPSP or any empirically supported educational/presurgical preparation interventions that (1) alert patients and parents of the early signs of CPSP or (2) provide behavioral management of symptoms. Patients seemingly “slip through the cracks,” with neither surgery nor anesthesia assuming responsibility for the prevention of CPSP or long-term care of patients who might be at risk for the development of CPSP.^31^

### Potential Solution

To identify those at risk for CPSP, the development and implementation of multicenter data repositories that include the administration of biobehavioral assays (e.g., bedside quantitative sensory testing, inflammatory/genetic markers, pain and emotional functioning questionnaires), which are both feasible to administer and predictive, are needed. Additionally, electronic health record (EHR) systems are promising tools but underutilized in pediatric clinical research.^32^ Given the amount of patient and provider data stored in EHRs coupled with advances in clinical research informatics tools, EHR data should be used to study and identify risk factors for the development of CPSP, with the ultimate goal of developing global clinical data research networks that have the capability of deep clinical phenotyping.^33^ Similarly, data mining techniques such as machine learning has been utilized to predict postsurgical opioid use^34,35^ and have also been proposed as a method to apply a systems biology framework to elucidate novel biological pathways involved in acute postoperative pain and CPSP, with a recent study using machine learning for targeted genetic profiling to explore CPSP risk in adult and pediatric patients.^36^

Additionally, appropriate presurgical preparation and education that target pain risk and coping would likely be important in the prevention of CPSP but to our knowledge do not exist. In general, presurgical preparation programs to prepare children for surgery, such as Meet Me at Mount Sinai,^37^ provide comprehensive emotional and cognitive preparation for surgery and have been found to decrease length of hospital stays, help with separation anxiety, and help with coping and sleep disturbance^37,38^; however, specific behavioral interventions targeting the prevention of CPSP in children are lacking. Studies of behavioral interventions in adults presenting for surgery are mixed, with a recent narrative review^39^ supporting the utility of relaxation, psychoeducation, and behavioral modification therapy but concluding that there is a need to strengthen the evidence of these interventions. Moving forward, there is a need to develop pediatric-focused behavioral interventions for the prevention of CPSP but also determine *how* and *why* existing evidence-based pain therapies work for certain patients and not others, which will be accomplished via mechanistic clinical trials and pharmacogenetics.

## State II: Perioperative Approaches

### Current Problem

During surgery, general anesthetics produce a state of drug-induced unconsciousness but not analgesia. The exception is ketamine, which produces dose-related unconsciousness and analgesia. Analgesics are administered according to weight-based dosing in response to clinical (patient movement) and autonomic (blood pressure, heart rate, respiratory rate, sweating) activity, rather than with a objective marker of nociception directly from the central nervous system. The mechanism and intensity of analgesia will vary with the class of drug, dosage, and route of administration. With respect to pain perception, a preclinical fMRI study in macaques found that noxious stimuli resulted in activation of the secondary somatosensory cortex and insula under propofol or pentobarbital anesthesia, whereas no activation was observed with isoflurane anesthesia.^40^ In humans, ongoing nociceptive processing has been shown to occur in adolescent patients under balanced general anesthesia.^41^

Nociceptive signaling to the brain during surgery may contribute significantly to perioperative stress and can have a profound effect in the perioperative period.^42,43^ Repeated nociceptive barrage can produce a condition of central sensitization where the brain becomes more sensitive to future stimuli that may be exacerbated by prior pain.^44–46^ This sensitization can impact the patient both during and after surgery and may necessitate increased postoperative pain control (e.g., increased medication).^47^ Central sensitization resulting from acute pain stimuli may contribute to the development of chronic neuropathic pain that occurs in 15% to 50% of all surgeries.^13^ Part of this may be as a result of an inability to measure pain load during surgery in an objective manner during surgery. As suggested previously it is, “not timing but duration and efficacy of an analgesic and antihyperalgesic intervention that are most important for treating pain and hyperalgesia after surgery.”^48(p551)^ The efficacy and sufficiently early administration of analgesia are of critical importance during the evacuation of wounded military personnel in the field, who would benefit tremendously from portable measures of pain and analgesia. As such, the ability to provide complete analgesia during surgery is a problem because no objective measures are routinely used to evaluate brain function during the surgical procedure.

What happens to central neural networks from surgical trauma during general anesthesia is not well understood. Animal and human imaging data suggest that ongoing nociceptive drive may continue following peripheral tissue damage, resulting in peripheral sensitization from nociceptive molecules such as bradykinin and neuroinflammatory changes.^49,50^ As a result of peripheral sensitization, the afferent barrage (including incomplete nerve conduction block with regional analgesic techniques) may continue. The analgesic status of a patient under general anesthesia is determined by weight-based dosing and the clinical and autonomic responses (patient movement, blood pressure, heart rate, respiratory rate, sweating) to noxious stimulation. The administration of muscle relaxants during anesthesia removes signs of inadequate analgesia such as patient movement and increased respiratory rate. With fMRI, spinal reflex responses, and somatosensory evoked potentials, Lichtner and colleagues^51^ showed that nociceptive activation in the spinal cord and brain of young adults persists during deep general anesthesia with propofol and remifentanil despite abolished clinical responses regarded as sufficient.^51^ Without an objective monitor of afferent nociceptive activity in C and A delta fibers, and even with the administration of analgesia, ongoing pain perception is likely in a significant number of patients under general anesthesia.

### Potential Solution

A solution for mitigating intraoperative effects of nociceptive barrage includes (1) defining patients at risk (see above) and treatment of amenable conditions (anxiety, depression, stress and preoperative pain), (2) aggressive intraoperative maintenance or prevention of nociceptive activity and unconscious perception of pain under anesthesia (a concept that is still evolving since the International Association for the Study of Pain definition of pain is in the conscious state), and (3) aggressive ongoing postoperative pain control. The importance of the presurgical brain state is probably underappreciated in routine surgeries. However, as is well documented in the literature, issues such as anxiety or depression, catastrophizing, and preoperative pain levels produce additional risks for the effects of afferent nociceptive barrage on neural networks that may be more easily sensitized or adversely affected by the surgical process (surgical trauma, stress, anesthetics, drugs including opioids, pain).^13,52–54^

**Figure 2.**
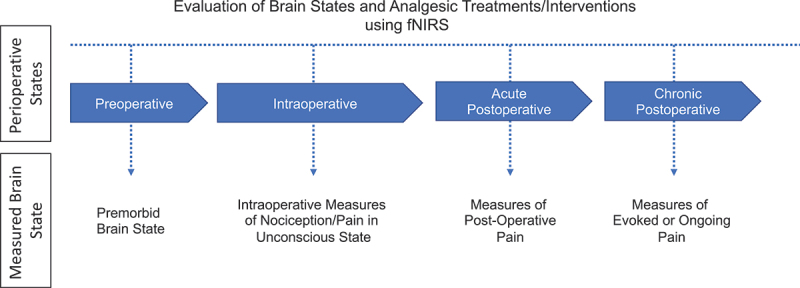
fNIRS-based brain measures of acute and/or ongoing pain measured during the various perioperative states. The measured brain states at each perioperative stage can be both intrinsic and extrinsic. *Intrinsic brain state* often refers to the brain in the absence of external stimuli, such as during persistent ongoing pain. In contrast, *extrinsic brain state* refers to the brain during an acute stimulus, intervention, or treatment.

A number of groups have been studying technologies for use as potential objective measures of pain in the operating room in an attempt to establish measures of analgesia in fully anesthetized patients.^55,56^ Our group has been evaluating a technology (functional near-infrared spectroscopy, fNIRS) for measures of nociception and pain in adult and pediatric populations during both awake and unconscious/anesthetized states.^41,57–59^ Low susceptibility to motion artifacts, flexibility in setup, and low overhead cost make fNIRS a useful neuro-investigative tool for a wide range of research and clinical applications involving pediatric patients. It is a popular technique to investigate typical and atypical development in infants,^60–62^ young children,^63,64^ and adolescents.^63,65,66^ If fully validated and successfully implemented in the operating room, fNIRS-based pain detection systems would allow for continual monitoring and maintenance of an adequate analgesic state in both pediatric and adult patients (Figure 2). We provide some details on the current status of the field and its potential for adoption in surgical practice and the utility for evaluation of current or new intraoperative analgesics (e.g., sodium channel blockers). We have based our fNIRS approach on evaluating signals from two main brain regions: the medial polar frontal cortex (mPFC) and the primary somatosensory cortex (SI). The mPFC is involved in higher-order pain processing such as perception and modulation,^67^ and the SI is the primary sensory region involved in nociception and chronic pain.^68,69^ The two regions respond in an inverse manner to pain/nociception (mPFC deactivates and SI activates), thereby providing a suitable anticorrelative marker. The premise of our approach to the use of fNIRS during surgery is based on a number of themes: (1) animal,^70^ including nonhuman primate^40^ and human data^58^ indicate that the pain pathways may be activated in the SI even under inhalational^71^ and propofol^72^ anesthesia; (2) surgical intervention, a controlled timed event, results in the potential initiation of chronic pain in a significant number of patients; (3) evaluation and response to pain during surgery are somewhat subjective and not based on specific/objective measures; and (4) an objective marker that allows for ongoing analgesia or immediate response to pain during the intra- and postoperative periods may provide an opportunity for diminished postoperative pain levels and risk of chronification (acute and chronic).

The challenge is to measure not only acute/evoked pain that may be produced by surgical procedures such as cutting, stretching, scraping cauterizing, etc., but also the magnitude of ongoing pain (intensity and duration). We have previously reported the use of fNIRS for evaluating evoked pain in fully anesthetized adolescent patients^58^ and adult patients undergoing sedation for colonoscopy.^73^ These signals are similar to, if not identical to, those measured in the awake state in healthy volunteers exposed to acute electrical or thermal pain.^74^ Though the frequency of evoked pain may contribute to or exacerbate central sensitization, the ongoing pain may be as important and more difficult to evaluate under general anesthesia. In order to accomplish this, we have established algorithms such as power spectral analysis^75^ and functional connectivity analysis that could gauge and measure ongoing pain in a manner similar to that previously reported by fMRI studies of chronic persistent pain.^76^ Improvements in these measures will allow us, we believe, to provide a measure of surgical pain load (SPL). Though having an objective measure of SPL using fNIRS or other imaging techniques may be useful in the context of the perisurgical period, the real question is whether decreasing SPL through improved analgesia will prevent or limit establishment or magnitude of CPSP.

Evaluation of intraoperative analgesics with an objective marker of evoked and ongoing pain could potentially have enormous benefits for inhibiting postoperative pain, in both the early and late course following surgery. For example, data supporting the use of sodium channel blockers during surgery provide a model for these and other drugs to truly evaluate their effects on the magnitude of blocking afferent nociceptive blockade on postoperative recovery^77^ (National Library of Medicine, NCT01907997, NCT01907997)^78^ or postoperative opioid consumption.^79^ Local anesthetics have been linked to inhibition of immune function.^80^ These are examples of having an objective metric of pain/nociceptive activation of brain systems that could have an enormous impact on both the perisurgical control of pain and long-term consequences of pain.

## State III: The Postoperative Imperative

### Current Problem

As already stated, the risk of developing CPSP is significant given the very limited understanding of the mechanisms contributing to CPSP and adequately providing intra- and postoperative analgesia. As pain evolves, it becomes increasingly difficult to treat and, unfortunately, a good model of brain rehabilitation in the postoperative period before pain chronifies does not exist. Additionally, though patients may be educated on immediate postoperative complications (e.g., severe pain, fever, bleeding), there is no standard practice that includes educating patients on the potential long-term complications such as signs and symptoms of neuropathic pain (e.g., burning, tingling, shooting, numbness, “pins and needles”).^81^

In cases when pain chronification has already occurred, treatments need to be employed early in the clinical course to help reverse or diminish chronic pain–associated comorbidities. One traditional treatment avenue has been opioids, which has resulted in a humanitarian crisis and are often ineffective in treating long-term pain.^82^ Specifically, research has found that over 80% of patients receive opioid prescriptions, often oxycodone or hydrocodone, after low-risk surgery,^80^ which are also the most commonly implicated in drug overdose deaths.^83^ Medical use of prescribed opioids during adolescence has also been found to be associated with later nonmedical opioid use in adulthood.^23^ Thus, finding alternatives to opioids in treating CPSP in adolescents is critical. However, the opioid crisis is attributed, in large part, to a dearth of research on how and why existing nonopioid pain therapies (e.g., behavioral interventions, anticonvulsants) work for certain patients and not others.^84^ A meta-analysis exploring the use of gabapentin for postsurgical pain in individuals 18 years or older concluded that though it improved the efficacy of opioids as well as reduced the need for analgesic consumption and opioid-related adverse effects, it is associated with side effects including sedation and dizziness, which is not ideal for an adolescent patient who has to focus at school.^85^ Additionally, it remains unclear whether gabapentin reduces mechanical hyperalgesia in and around the wound, and studies on dose–response efficacy are lacking.^85^ One randomized clinical trial (RCT) in adults found that compared to placebo, gabapentin use may promote opioid cessation postsurgery and decrease the duration of postoperative opioid use, but it did not have an impact on time to pain cessation.^86^ Further, several RCTs on the use of gabapentin in pediatric surgical samples demonstrated some benefits for its use as an adjunct to improve pain control; however, overall, it did not help with opioid-related side effects. Additionally, its effects on long-term pain prevention or treatment are unclear.^87–89^

Though there is robust evidence for the use of psychological treatments, especially cognitive behavioral therapy (CBT), for the treatment of chronic pain in children,^90–96^ little has been conducted specifically on the use of behavioral interventions for the prevention and/or treatment of CPSP in children and adolescents.^97^ An innovative interdisciplinary program for adults at Toronto General Hospital, the Transitional Pain Service, focuses on CPSP prevention and treatment and involves intensive perioperative psychological, physical, and pharmacological management and has demonstrated strong preliminary results from two nonrandomized, clinical practice–based trials.^98,99^ Specifically, the Transitional Pain Service utilizes an acceptance and commitment therapy (ACT) approach and has found improvements in pain, pain interference, pain catastrophizing, symptoms of anxiety and depression, and opioid use. Other studies in adults also support the use of ACT in the treatment of CPSP. Specifically, ACT, known as third-wave CBT, aims to address avoidance behaviors by increasing openness to difficult experiences such as pain and to facilitate behavior change processes that are in accord with living a values-based life.^100^ A pilot randomized control study found that among veterans presenting for orthopedic surgery, participants who completed an ACT workshop reached pain and opioid cessation sooner than those in the treatment as usual group.^101^ Similar programs and RCTs are needed in pediatric surgical populations; however, preliminary data show support for the utility of ACT in treating pediatric chronic pain.^102^ Specifically, Wicksell and colleagues published the first ACT RCT for pediatric pain and found that when compared to a multidisciplinary treatment approach, which included amitriptyline medication, youth who participated in a 10-week ACT intervention demonstrated substantial and sustained improvements in fear of pain, pain interference, and quality of life.^103^ A more recent small RCT comparing an ACT-based treatment with a control condition in children ages 7 to 12 with chronic pain found significantly greater improvements in functional disability at the end of treatment and at 3.5 and 6.5 months posttreatment in the ACT group compared to the controls.^104^

### Potential Solution

More programs and RCTs that focus on the prevention and treatment of CPSP in children and adolescents are sorely needed. First, patients and their parents need to be made aware of the signs and symptoms of neuropathic pain so they can seek help at their onset. This could be implemented by the preoperative clinic and reviewed at surgery discharge and follow-up visits. Additionally, as Borsook^105^ noted nearly a decade ago, though our understanding of chronic pain has evolved significantly, new scientific approaches to successfully developing effective medications are lacking. This has not changed in the 10 years since that article was published.^105^ There is a need for the development of widely available, affordable analgesics for chronic pain that surpass the efficacy of existing treatments; have fewer side effects, including addiction; and modify disease in a way that is predictive and adaptive.^105^ A better understanding of the mechanisms contributing to CPSP could help with this endeavor. Further, behavioral interventions, such as CBT and ACT, need to be tailored to meet the needs of young patients at risk for and/or living with CPSP. However, we need to go a step further and actually conduct mechanistic clinical trials to elucidate the biological or behavioral process, the pathophysiology of a disease, or the mechanism of action of an intervention in order to understand treatment response and enhance our capability for developing individually tailored patient-oriented interventions.^106^

## Conclusion

CPSP is a significant humanitarian burden, and there is a paucity of research or effective interventions to prevent and/or treat this problem in children and adolescents. An improved comprehension of the biobehavioral and neural mechanisms linked to CPSP will provide finer tools for optimizing the selection of treatments for individual patients. Moreover, data that demonstrate the underlying pathobiological pain mechanism(s) active in CPSP, particularly those nonresponsive to current therapies, may be used to validate novel strategies, both pharmacological and nonpharmacological. Ultimately, the goal of future research in pediatric CPSP should be to (1) enhance our understanding of the neurobiology of CPSP, (2) provide a metric to follow patients with CPSP in the clinic, and (3) provide a metric for those who are at greatest risk of chronification. However, these goals are not simple, nor will they be accomplished easily. Barriers to achieving the goals of our proposed solutions to preventing and treating pediatric CPSP include (1) identifying clearly defined biomarkers that are sensitive and reproducible, which requires significant research funding and time; (2) acceptance by surgeons and anesthesiologists of the importance of measuring nociception during surgery, which may be difficult in managed care settings; (3) outcome studies showing the benefits of measuring intraoperative pain load as a correlation of the development of chronic pain; and (4) understanding the cross-country and cross-cultural variation in analgesic prescribing following surgery, which is currently poorly understood.^107^ At the heart of scientific endeavor is interdisciplinary collaboration, which can be time-consuming and expensive; however, the benefits of collaborating (e.g., surgeons, anesthesiologists, pain clinicians, neuroscientists, basic scientists, behavioral scientists, and epidemiologists) could transform the field of pediatric CPSP and eliminate significant suffering by informing earlier risks for the development of CPSP and providing more personalized and precise treatment for those affected by CPSP.
